# Risk Stratification in Advanced Biliary Tract Cancer: Validation of the A.L.A.N. Score

**DOI:** 10.1155/2020/6180613

**Published:** 2020-06-23

**Authors:** Lukas Müller, Aline Mähringer-Kunz, Florian Jungmann, Yasemin Tanyildizi, Fabian Bartsch, Carolin Czauderna, Christoph Düber, Peter R. Galle, Arndt Weinmann, Roman Kloeckner, Felix Hahn

**Affiliations:** ^1^Department of Diagnostic and Interventional Radiology, University Medical Center of the Johannes Gutenberg-University Mainz, Mainz, Germany; ^2^Department of Neuroradiology, University Medical Center of the Johannes Gutenberg-University Mainz, Mainz, Germany; ^3^Department of General, Vascular, and Transplant Surgery, University Medical Center of the Johannes Gutenberg-University Mainz, Mainz, Germany; ^4^Department of Internal Medicine I, University Medical Center of the Johannes Gutenberg-University Mainz, Mainz, Germany; ^5^Clinical Registry Unit (CRU), Department of Internal Medicine, University Medical Center of the Johannes Gutenberg-University Mainz, Mainz, Germany

## Abstract

**Background:**

In addition to the clinical parameters, immune-inflammatory markers have emerged as prognostic factors in patients with advanced biliary tract cancer (ABC). The recently proposed A.L.A.N. score combines both in an easily applicable manner. The aim of this study was to perform the first external evaluation of this score.

**Methods:**

All patients from our clinical registry unit who had unresectable ABC underwent first-line chemotherapy from 2006 to 2018 and met the inclusion criteria of the original study were included (*n* =  74). The A.L.A.N. score comprises the following parameters: actual neutrophil count, lymphocyte-to-monocyte ratio, albumin, and neutrophil-to-lymphocyte ratio (A.L.A.N.). Univariate and multivariate hazard regression analyses were performed to evaluate the score's parameters regarding overall survival (OS). The concordance index (C-index) and integrated Brier score (IBS) were calculated to evaluate the score's predictive performance.

**Results:**

Low, intermediate, and high A.L.A.N. scores corresponded to median OS of 21.9, 11.4, and 4.3 months, respectively, resulting in a significant risk stratification (log-rank *p*=0.017). In multivariate analysis, a high-risk A.L.A.N. score remained an independent predictor of poor survival (*p*=0.016). Neutrophil-to-lymphocyte ratio was not a significant factor for poor OS in the analyses in the cohort. The score's ability to predict individual patient survival was only moderate with a C-index of 0.63.

**Conclusions:**

The A.L.A.N. score can be used to identify risk groups with a poor prognosis prior to the start of chemotherapy. However, the ability of the score to predict individual patient outcome was only moderate; thus, it may only serve as a minor component in the complex interdisciplinary discussion.

## 1. Introduction

Biliary tract cancer consists of a group of heterogeneous cancer entities deriving from the biliary system, including intrahepatic cholangiocarcinoma (iCCA), perihilar cholangiocarcinoma (pCCA), distal cholangiocarcinoma (dCCA), and gallbladder carcinoma (GBC) [1]. The incidence of biliary tract cancer, which accounts for 3% of all gastrointestinal cancer cases, is relatively low in Western countries, with a range of 0.35–2/100,000 annually [2, 3]. A rising trend in iCCA is reported, while incidence rates for extrahepatic CCA remain constant or even show a decrease [4]. However, the increase in iCCA cases is likely influenced by an improvement in diagnosis due to better imaging and diagnostic techniques and by prior misclassification (diagnostic transfer) [5, 6].

Resection remains the only curative option, but it is only available for less than one third of the patients and the majority of patients are diagnosed in advanced stages (advanced biliary tract cancer, ABC) [7, 8]. Moreover, even when biliary tract cancers are suspected early on, imaging evaluation especially of nonmass forming CCAs is challenging [9, 10]. For patients with metastatic or locally advanced disease, chemotherapy is the mainstay of therapy [11]. In the first-line treatment, the regimes are mainly gemcitabine-based, and gemcitabine/cisplatin is the most common combination since publication of the UK-ABC 02 trial results [11]. However, the option of multiple chemotherapy cycles, necessary for an appropriate response, is often limited by high toxicities and requires good reserves of renal and liver function [12, 13]. Considering the poor prognosis of patients with ABC and the potential side effects of aggressive chemotherapy, a risk score providing a priori estimate of survival might have a direct impact on the patient's assessment regarding treatment options.

Several risk factors correlated to overall survival (OS) have emerged in recent studies and led to different stratification approaches, including risk factors like primary tumour location, disease status, metastatic sites, Eastern Cooperative Oncology Group (ECOG) performance status (PS), and biochemical laboratory parameters [14–18]. Moreover, the immune system is increasingly recognized as important [19, 20]: neutrophil, lymphocyte, or monocyte counts and their ratios functioned as predictors for median OS of patients suffering from ABC [14, 21–24]. Recently, Salati et al. proposed their A.L.A.N. (actual neutrophil count, lymphocyte-to-monocyte ratio, albumin, neutrophil-to-lymphocyte ratio) score (for ABC patients receiving first-line chemotherapy [25]. This score combined one function-related biochemical parameter with three immune-inflammatory markers and resulted in a significant stratification regarding OS in their cohorts. All the included immune-inflammatory markers emerged as independent prognostic factors. In particular, this was the first study to show a predictive value of the lymphocyte-to-monocyte ratio for ABC patients.

However, even though the score showed promising results in its original publication, external validation of the A.L.A.N. score is still lacking and is mandatory before the score can be implemented in clinical practice. To the best of our knowledge, no attempt has been made to tackle this issue. Therefore, the aim of this study was to perform the first external validation of the A.L.A.N. score.

## 2. Materials and Methods

### 2.1. Patient Selection

The study was approved by the responsible ethics committee for the retrospective analysis of clinical data (permit number: 2018–13618). For primary data collection, all patients with biliary tract cancer treated at our tertiary care centre were identified with the help of our clinical registry unit (CRU). The development of the study was based on the criteria of the TRIPOD statement [26].

To ensure comparability, the inclusion and exclusion criteria were adopted from the original A.L.A.N. publication [25]: all patients with histopathologically confirmed unresectable ABC undergoing first-line chemotherapy were retrospectively analysed. Patients with mixed hepatocellular-cholangiocellular and ampullary carcinoma were excluded. Furthermore, none of the patients received locoregional therapy of the primary tumour or surgery/ablation of the metastatic sites. The baseline parameters before the first chemotherapy cycle, including demographic data, performance status (PS), primary tumour site, disease status, and chemotherapy regimen were derived from the CRU. Missing baseline parameters led to exclusion. All laboratory parameters including haematological and biochemical parameters were gathered from the central laboratory information system.

### 2.2. Calculation of the A.L.A.N. Score

The A.L.A.N. score is calculated by the summed score of the following variables: actual neutrophil count (ANC) (≤8000/*μ*l, 0 points; >8000/*μ*l, 1 point), lymphocyte-to-monocyte ratio (LMR) (≥2.1, 0 points; <2.1, 1 point), albumin (≥3.5 g/dl, 0 points; <3.5 g/dl, 1 point), and neutrophil-to-lymphocyte ratio (NLR) (≤3.0, 0 point; >3.0, 1 point). For further risk stratification, three risk groups are defined: 0 points, low risk; 1–2 points, intermediate risk; and 3–4 points, high risk.

### 2.3. Statistical Analysis

Statistical analysis and graphics design were performed in *R* 3.5.1 (A Language and Environment for Statistical Computing, *R* Foundation for Statistical Computing, http://www.R-project.org; accessed 2019).

Categorical and binary baseline parameters were reported as absolute numbers and percentages. Continuous data were reported as median plus range. Thresholds for dichotomisation of the laboratory parameters were adapted from the original A.L.A.N. study. The OS as primary end-point was calculated from the start of the first-line chemotherapy to the date of death or last contact in followup. Kaplan–Meier curves were created with the packages “survminer” and “survival” (https://cran.r-project.org/package=survminer, https://CRAN.R-project.org/package=survival, accessed on Oct 31, 2019) and strata compared with the log-rank test. The effect of the risk stratification, as well as an evaluation of the included factors, was performed with multivariate Cox proportional-hazards regression models to assess hazard ratios (HR) and corresponding 95% confidence intervals (CIs). For further validation of the score, Harrell's C concordance index (C-index) was calculated by using the “Hmisc” package (https://cran.r-project.org/package=Hmisc, accessed Oct 31, 2019). The C-index ranges from 0 to 1, where 0.5 indicates no predictive ability and 1 indicates perfect predictive ability [27]. Prediction error curves were based on the Brier score (package “pec,” https://cran.r-project.org/package=pec, accessed on Oct 31, 2019) [28]. The Brier score at time *t* is the mean squared difference between the observed outcome (1 for event and 0 otherwise) and the predicted outcome probability at time *t*. The integrated Brier score (IBS) over the interval [0 months, 48 months] was calculated as a summary measure of prediction error. A *p* value of <0.05 was considered statistically significant for all tests.

## 3. Results

### 3.1. Patient Recruitment

**Figure 1:**
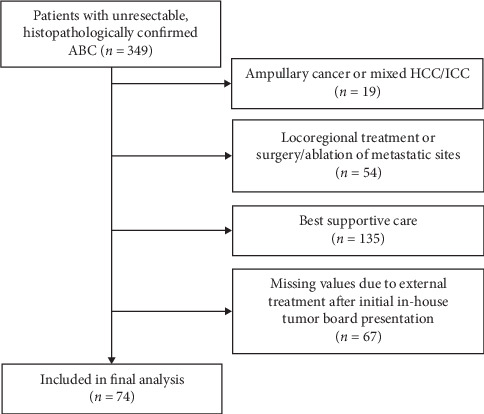
Strobe flow diagram showing the number of patients included in the final analysis and the reasons for dropout. ABC: advanced biliary tract cancer; HCC/ICC: mixed hepatocellular-cholangiocellular carcinoma.

From our CRU, we extracted a total of 349 patients with unresectable ABC, who were treated between January 2006 and June 2018. A total of 275 patients had to be excluded, leaving 74 patients included in the final analysis (Figure 1).

The median age of our cohort was 65 years (range: 22–86 years). A total of 32 (43%) patients were female. Primary tumour sites were iCCA (*n* = 49, 66%), pCCA (*n* = 11, 15%), dCCA (*n* = 3, 4%), and GBC (*n* = 11, 15%).  Table 1 provides the baseline patient characteristics of our cohort compared with both original A.L.A.N. cohorts.

The median OS of our patient cohort was 9.0 months (95% CI 6.0–13.2 months) and the 1-year OS was 38%.

### 3.2. A.L.A.N. Score

**Figure 2:**
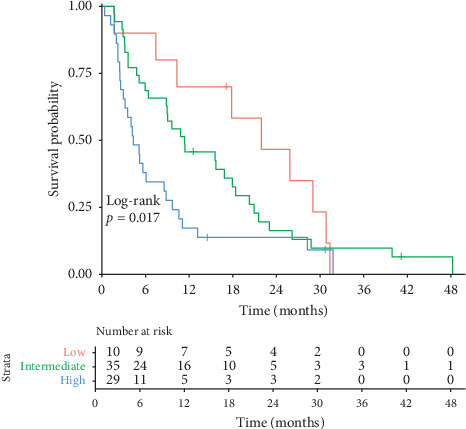
Kaplan–Meier curves of OS, beginning with start of first-line chemotherapy for ABC patients and stratified according to the proposed A.L.A.N. score risk groups (low risk: red; intermediate risk: green; high risk: blue).

Of the 74 patients, 10 (13.5%) were in the low-risk A.L.A.N. group (score 0), 35 (47.3%) in the intermediate-risk group (score 1-2), and 29 (39.2%) in the high-risk group (score 3-4). The median OS was 21.9 months in the low-risk group, 11.4 months in the intermediate-risk group, and 4.3 months in the high-risk group. The Kaplan–Meier curves are shown in Figure 2.

A comparative presentation of the median survival rates for our study and the original A.L.A.N. study including survival times and confidence intervals is provided in Table 2.

**Figure 3:**
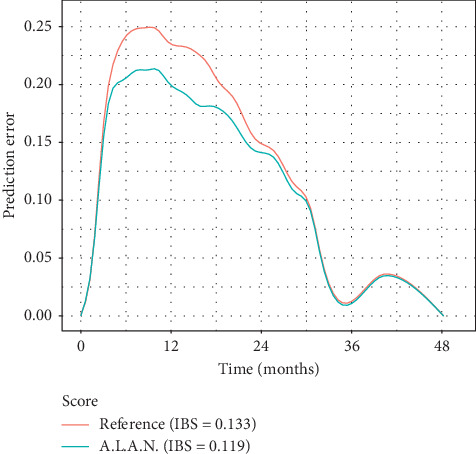
Prediction error curves and integrated Brier score (IBS) for Kaplan–Meier estimates based on the A.L.A.N. stratification and on the Kaplan–Meier estimates for all patients without any stratification (reference).

Harrell's C-index was 0.63. Prediction error curves are shown in Figure 3. The IBS for the A.L.A.N. score was 0.119 for the first 48 months, compared to an IBS of 0.133 for the unstratified cohort. As indicated by the prediction errors, the A.L.A.N. score confers a survival discrimination, particularly in the period ranging from 6 to 18 months after initiation of chemotherapy (see Figure 2).

**Figure 4:**
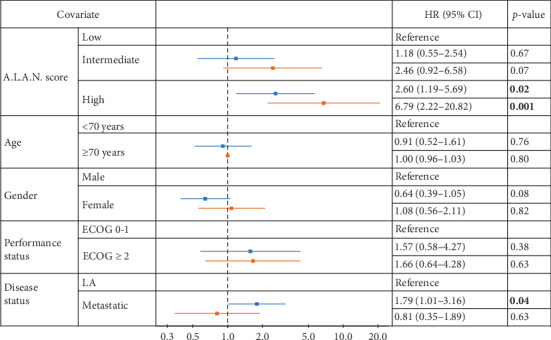
Results of multivariate analysis of the A.L.A.N. score and other risk factors for our (blue) and the original A.L.A.N. validation cohort (red) [25]. ECOG: Eastern Cooperative Oncology Group; LA: locally advanced; HR: hazard ratio; CI: confidence interval. Significant *p* values depicted in bold.

To compare these results with the original study, a Cox regression model including A.L.A.N. score risk groups, age, gender, performance status, and disease status was used. A high-risk A.L.A.N. score was an independent prognostic factor (HR = 2.60, *p*=0.016). Furthermore, the disease status had a significant prognostic impact (HR = 1.79, *p*=0.045). However, an intermediate-risk A.L.A.N. score, age, gender, and performance status had no additional predictive value (Figure 4).

In univariate analysis, ANC (*p*=0.003), LMR (*p*=0.013), and albumin (*p*=0.045) had a significant influence on median OS (Table 3). However, in a Cox regression including ANC, LMR, albumin, and NLR (A.L.A.N.), only ANC (HR = 2.2, *p*=0.003) remained an independent prognostic factor.

## 4. Discussion

To the best of our knowledge, this is the first study to perform an external validation of the recently published A.L.A.N. score for risk stratification of ABC patients receiving first-line chemotherapy. In our cohort, a higher A.L.A.N. score was associated with an increased hazard, and risk stratification yielded median OS of 21.9, 11.4, and 4.3 months for low-risk, intermediate-risk, and high-risk patients, respectively. However, the ability of the score to predict individual patient outcomes was only moderate, as indicated by a Harrell's C-index of 0.63.

To ensure comparability with the original study, we included the same risk factors in a multivariate model [25]. A high A.L.A.N. score and the disease status were independent prognostic factors for median OS. This accords with previously published results: patients with metastatic disease typically have worse prognoses than patients with locally advanced disease [14, 15, 29].

In contrast to prior publications, the performance status was not associated with an increased risk of poor survival in the original study or in our external validation [14, 15, 25, 30]. For our cohort, the main reason for this result might be the low number of patients with ECOG status 2 or higher (*n* = 5). This may be due to selection bias, as patients with a poor ECOG status are more likely to receive the best supportive care compared with a systemic treatment [31]. However, a larger variety of systemic therapy options and a better understanding of the side effects have led to higher treatment rates among more afflicted patients in recent years [32].

Even though several stratification systems for patients with ABC have been developed [14–16], none has been established in daily clinical practice. Salati et al. used a novel approach and included immune-related markers in their recently proposed risk score.

Of the included immune-inflammatory factors, ANC and LMR showed an influence on OS in univariate analysis. However, ANC remained the only independent risk factor in multivariate analysis. The original A.L.A.N. study was the first to show an independent prognostic effect of the LMR on ABC patients. Our results did not confirm these findings. Contrary to our expectations, a low albumin serum level showed a significant influence on median OS only in univariate analysis and lost its predictive value in multivariate analysis [33, 34]. The univariate analysis of the original A.L.A.N. validation cohort also demonstrated no significance for albumin as a prognostic marker. In contrast to previously reported results, the influence of NLR could not be confirmed in this study [21, 35]. This might be at least partly due to the moderate sample size, and the cut-off used for NLR (NLR > 3.0) might not be optimal for our cohort.

Regarding cut-offs used for stratification, one difficulty new prediction scores face is the risk of “overfitting.” In general, this is described as “a phenomenon occurring when a model maximizes its performance on some set of data, but its predictive performance is not confirmed elsewhere due to random fluctuations of patients' characteristics in different clinical and demographical backgrounds [36].” The low number of patients with ABC, especially, might increase the influence of this effect, as general representation is hard to attain within a single-centre cohort. However, in our cohort, the median OS times according to the three A.L.A.N. risk groups were 21.9, 11.4, and 4.3 months, corresponding extremely well with the median OS of the original A.L.A.N. exploratory cohort (22, 12, and 5 months). Thus, overfitting was not observed as a limiting factor.

However, even though risk stratification according to the A.L.A.N. score resulted in significant divergence of Kaplan–Meier curves in our cohort, concordance index calculation provides the probability that a randomly selected patient who experienced an event (in our case, death) had a higher risk score than a patient who had not experienced the event. Therefore, it can be regarded as a predictive measure of the individual patient outcome. Due to the deaths in all risk groups during the observation period, the discriminative ability of the score to predict which patient would die first when patients were randomly selected was only moderate in our study. Consequently, therapeutic implications from the score on an individual level have to be drawn with caution. This fact has repeatedly been observed in previous evaluations of risk scores [37, 38].

Furthermore, as chemotherapy is the mainstay of treatment for ABC patients and carries a survival advantage compared with the best supportive care, in clinical practice, chemotherapy is started if deemed oncologically reasonable, regardless of certain scoring values [39]. Clear-cut decision-making based only on scores is therefore virtually impossible, and currently no predictive system can replace the decision of an interdisciplinary tumour board.

The different ratio of patients treated with gemcitabine/cisplatin in our cohort compared with the original A.L.A.N. cohort should also be considered. Gemcitabine/cisplatin has been the first-line chemotherapy since 2010, following the results of the UK-ABC 02 trial in that year [11]. Adherence to this regime since then might have improved the patients' outcomes. As our observation period started in 2006, the percentage of patients receiving therapies other than gemcitabine/cisplatin is therefore higher compared with the original A.L.A.N. cohorts (60% vs. 42% in the exploratory cohort and 45% in the validation cohort). However, as the A.L.A.N. score is not created for a distinct regime, we included all suitable patients, independent of their first-line chemotherapy, to better reflect a real-world situation.

Our analysis is limited by several factors. First and most important, the data acquisition was retrospective and based on the patients of a single centre. Second, the sample size was only moderate (*n* = 74). However, due to the rare incidence and the strict inclusion criteria, our patient cohort was larger than the validation cohort of the original A.L.A.N. study. Moreover, current studies investigating new therapeutic options for ABC patients, for example, the combination of chemotherapy with locoregional treatment, radiation, or targeted and immune therapy, lead to a further diversification of treatment [40–42] and increase interstudy heterogeneity of the patient cohorts. We deliberately did not perform any kind of imputation of missing values and included only patients with the complete data needed for the calculation of the score and comparison with the original A.L.A.N. cohort. This reduced the patients' numbers and statistical power in favour of data completeness.

## 5. Conclusions

The A.L.A.N. score with its easily applicable parameters was able to differentiate between low-, intermediate-, and high-risk patients, and it can be used to identify risk groups with a poor prognosis prior to the start of chemotherapy. However, the ability of the score to predict individual patient outcome was only moderate; thus, it may only serve as a minor component in the complex interdisciplinary discussion. As chemotherapy is the only therapy option for patients with ABC, no patient for whom chemotherapy is deemed oncologically reasonable should be excluded due to A.L.A.N. score alone.

## Figures and Tables

**Table 1:** Baseline characteristics of the patients in this study and in the original A.L.A.N. study^*∗*^.

	This study	Original A.L.A.N. exploratory cohort	Original A.L.A.N. validation cohort
	(*n* = 74)	(*n* = 123)	(*n* = 60)
Age, years (median, range)	65 (22–86)	67 (29–85)	64 (54–70)
*Gender*			
Female	32 (43%)	65 (53%)	31 (52%)
Male	42 (57%)	58 (47%)	29 (48%)

*Performance status*			
ECOG 0-1	69 (93%)	101 (82%)	50 (83%)
ECOG ≥2	5 (7%)	22 (18%)	10 (17%)

*Primary tumour site*			
iCCA	49 (66%)	61 (50%)	17 (28%)
pCCA	11 (15%)	15 (12%)	18 (30%)
dCCA	3 (4%)	9 (7%)	19 (32%)
GBC	11 (15%)	38 (31%)	0
Unknown	0	0	13 (20%)

*Cirrhosis among patients with iCCA*			
Yes	7 (14%)	5 (8%)	—
No	42 (86%)	56 (92%)	—

*Disease status*			
Locally advanced	24 (32%)	15 (12%)	15 (25%)
Metastatic	50 (68%)	108 (88%)	45 (75%)

*First-line chemotherapy*			
Gemcitabine and cisplatin	30 (40%)	71 (58%)	33 (55%)
Gemcitabine	16 (22%)	8 (6%)	13 (22%)
Others^†^	28 (38%)	44 (36%)	14 (23%)

*Combined agents*			
Yes	53 (72%)	110 (89%)	—

No	21 (28%)	13 (11%)	—
*Second-line chemotherapy* ^††^			
Yes	23 (31%)	36 (29%)	24 (40%)
No	51 (69%)	87 (71%)	36 (60%)

*Laboratory test (median, range)*			
ANC (cells/*μ*l)	6012 (1867–15548)	5504 (1690–36230)	—
LMR	2.19 (0.80–66.83)	—	—
Albumin (g/dl)	3.5 (1.7–4.6)	3.7 (2.1–4.9)	—
NLR	4.87 (1.11–12.80)	—	—

ECOG: Eastern Cooperative Oncology Group; iCCA: intrahepatic cholangiocarcinoma; pCCA: perihilar cholangiocarcinoma; dCCA: distal cholangiocarcinoma; GBC: gallbladder cancer; ANC: actual neutrophil count; LMR: lymphocyte-to-monocyte ratio; NLR: neutrophil-to-lymphocyte ratio. ^*∗*^Data for the A.L.A.N. cohorts are adapted from the original publication and presented for comparison [25]. ^†^Other chemotherapy regimens for our cohort were: gemcitabine and sorafenib (*n* = 12); capecitabine and oxaliplatin (*n* = 5); capecitabine (*n* = 3); gemcitabine and oxaliplatin (*n* = 2); fluorouracil and imantinib (*n* = 2); fluorouracil, folinic acid, and irinotecan (*n* = 1); cisplatin and fluorouracil (*n* = 1); irinotecan (*n* = 1); oxaliplatin (*n* = 1). ^††^As further treatment during the course of disease.

**Table 2:** Comparison of median OS among the A.L.A.N. subgroups.

A.L.A.N. subgroups	Low risk (0 points)	Intermediate risk (1–2 points)	High risk (3-4 points)	*p* value
This study: median OS (95% CI), m	21.9 (10.3–30.9)	11.4 (6.4–18.0)	4.3 (3.0–8.6)	**0.017**
Original A.L.A.N. exploratory cohort: median OS (95% CI), m	22 (14–32)	12 (8–15)	5 (2–8)	**<0.001**
Original A.L.A.N. validation cohort: median OS (95% CI), m	12.9 (8.7–26.4)	9.3 (7.4–14.7)	4.3 (2.6–9.2)	**0.005**

OS: overall survival; CI: confidence interval; m: months. Significant *p* values are depicted in bold.

**Table 3:** Univariate and multivariate analysis of the A.L.A.N. score factors.

Analysis	Univariate			Multivariate		
Covariate	HR	95% CI	*p* value	HR	95% CI	*p* value
*ANC*						
>8000	2.19	1.30–3.70	**0.003**	2.23	1.31–3.78	**0.003**

*LMR*						
<2.1	1.87	1.14–3.07	**0.013**	1.62	0.86–3.08	0.137

*Albumin (g/dl)*						
<3.5	1.65	1.01–2.69	**0.045**	1.27	0.67–2.39	0.459

*NLR*						
>3	1.66	0.88–3.13	0.118			

ANC: actual neutrophil count; LMR: lymphocytes-monocytes ratio; NLR: neutrophil-lymphocytes ratio; HR: hazard ratio; CI: confidence interval. Significant *p* values depicted in bold.

## Data Availability

The data that support the findings of this study are included within the article. The primary data are stored in internal clinical registry software specially developed for the clinical characterization of patients with HCC and CCC to ensure participant confidentiality. The datasets used and analysed during the current study are available from the corresponding author upon reasonable request.
